# Unraveling the dual threat: sarcopenia and insufficient physical activity in diabetes risk

**DOI:** 10.3389/fendo.2024.1507657

**Published:** 2025-01-08

**Authors:** Hui Shi

**Affiliations:** Department of Clinical Nutrition, Zibo First Hospital, Zibo, China

**Keywords:** diabetes, sarcopenia, physical activity, NHANES, population structure

## Abstract

**Purpose:**

This study aimed to investigate the alterations in diabetes risk associated with sarcopenia and insufficient physical activity, as well as the demographic shifts within the diabetic population.

**Method:**

Utilizing pertinent data from the National Health and Nutrition Examination Survey (NHANES) database spanning 2011 to 2018, the criteria for sarcopenia were established by the Foundation for the National Institutes of Health. These criteria were calculated using lean body mass data in conjunction with body mass index data. Physical activity levels were assessed using the PAQ questionnaire from the NHANES database. The presence of diabetes was determined through the DIQ questionnaire and the laboratory examination within the NHANES database. The analysis was performed using multivariable logistic regression.

**Result:**

The prevalence of both sarcopenia and insufficient physical activity in the diabetic population was 188% greater than in the non-diabetic population. Sarcopenia and insufficient physical activity were positively correlated with an increased risk of diabetes onset, demonstrating a 1.45-fold heightened risk when both conditions were present (OR=2.45,95%CI,1.35-4.44,P<0.05). This combined effect was significantly greater than the risk associated with sarcopenia alone (OR=1.84,95%CI,1.09-3.11,P<0.05) or insufficient physical activity alone (OR=1.55,95%CI,1.11-2.15,P<0.05).

**Conclusion:**

A synergistic relationship exists between sarcopenia and insufficient physical activity, resulting in a markedly elevated risk of diabetes when both conditions are present concurrently. Therefore, comprehensive diabetes management strategies should prioritize populations exhibiting both sarcopenia and insufficient physical activity.

## Introduction

Diabetes is a global public health problem, with an estimated global prevalence of diabetes in people aged 20-79 years being 10.5% (536.6 million) in 2021 and rising to 12.2% (783.2 million) by 2045 (1). Global diabetes-related health expenditure was estimated at $966 billion in 2021 and is projected to reach $1054 billion by 2045 (1). Diabetes is closely associated with many diseases, leading to organ function damage and even failure (2–4). Despite extensive research and efforts dedicated to understanding the pathogenesis, developing medications, and implementing management strategies for diabetes (5–7), it remains challenging to curtail its increasing prevalence (8).

Sarcopenia has emerged as a condition of significant interest in recent years, primarily characterized by a reduction in muscle mass, strength and function (9). According to the European Working Group on Sarcopenia in Older People, sarcopenia is defined as “a syndrome characterized by progressive and systemic loss of skeletal muscle mass and strength, with an increased risk of adverse outcomes such as physical disability, poor quality of life, and mortality (10).” It is conservatively estimated that sarcopenia currently affects approximately 50 million individuals, with projections suggesting this number could rise to 200 million over the next four decades (11). Sarcopenia results in a reduction of muscle mass, thereby compromising blood glucose regulation (12). From this perspective, a significant association between sarcopenia and diabetes is evident. Furthermore, research has demonstrated that sarcopenia is independently correlated with type 2 diabetes (13). Insufficient physical activity also constitutes a risk factor for diabetes (14, 15). Empirical studies have indicated that insufficient exercise adversely affects insulin sensitivity (16), which can subsequently diminish the body’s capacity to process blood glucose effectively and potentially precipitate the onset of diabetes.

While many studies have examined how sarcopenia and insufficient physical activity individually affect diabetes (17, 18),the combined impact remains unclear. This paper aims to investigate the joint effect of sarcopenia and insufficient physical activity on diabetes risk using population-based data.

## Materials and methods

### Data source

Publicly accessible data files from the National Health and Nutrition Examination Survey (NHANES) database were utilized in this study. NHANES is a population-based, cross-sectional survey that encompasses interviews, physical examinations, and laboratory assessments. The primary objective of NHANES is to evaluate the nutritional status of the United States’ civilian, non-institutionalized population and to explore its relationship with health promotion and disease prevention. The data are released biennially and are derived from a nationally representative sample, employing a multi-stage probability sampling design and weighting methodology. NHANES is scheduled for an annual review by the Ethics Review Board of the National Center for Health Statistics to ensure adherence to ethical and scientific standards (19).

### Study population

**Figure 1:**
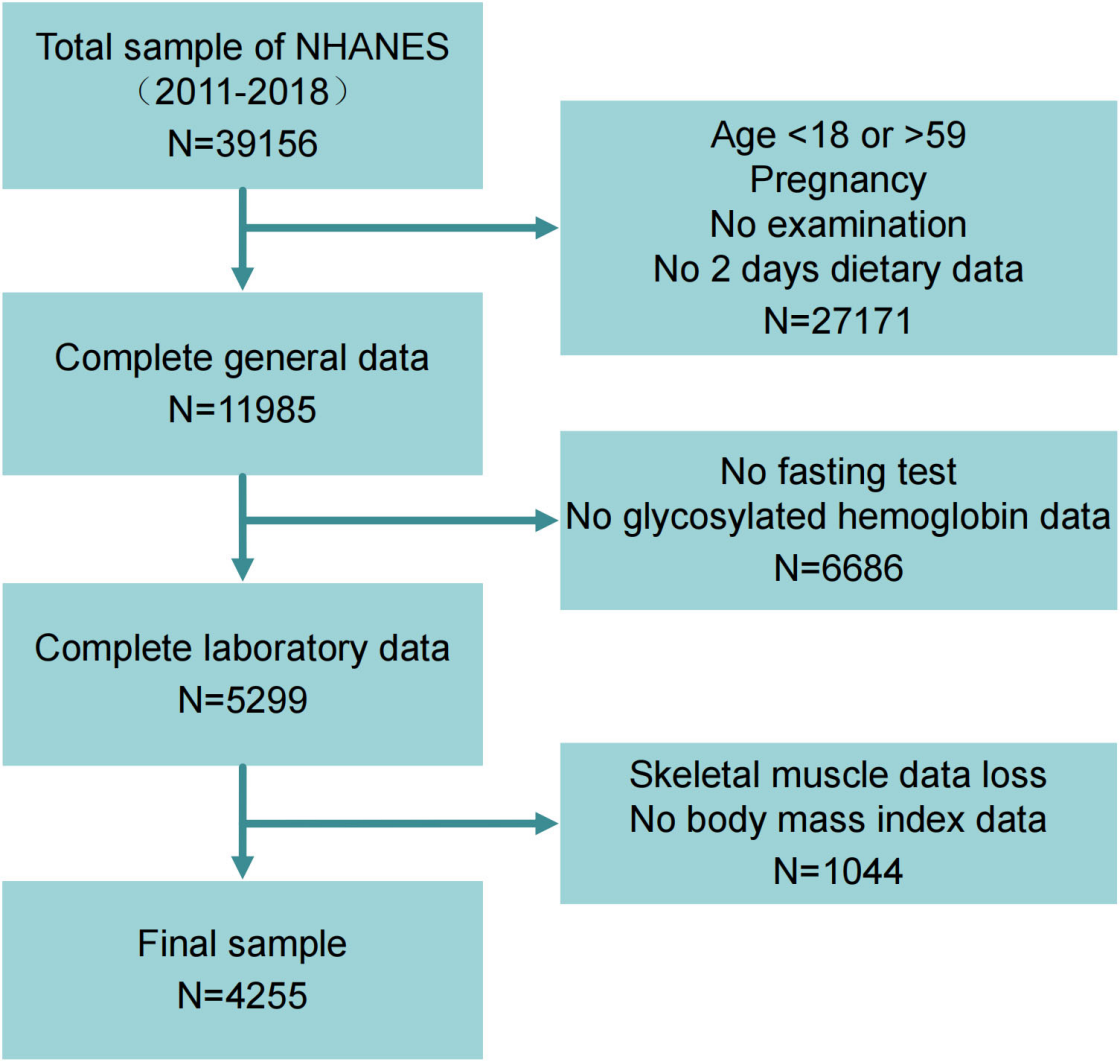
Flowchart for choosing target population.

In this study, data from 39,156 samples were utilized from the NHANES database spanning the 2011-2012, 2013-2014, 2015-2016, and 2017-2018 cycles. The samples were screened according to the study’s requirements, with exclusion criteria as follows: (1) individuals aged under 18 or over 59, pregnant individuals, those without examinations, and those lacking two days of dietary data (N=27,171); (2) individuals without fasting test results or glycosylated hemoglobin data (N=6,686); (3) individuals with missing skeletal muscle data or body mass index data (N=1,044). Ultimately, 4,255 participants met the eligibility criteria for inclusion in the study. **Figure 1** illustrates the screening process.

### Diagnosis of diabetes

In the DIQ questionnaire, being told by the doctor that “having diabetes” was the main criterion, fasting blood glucose ≥126 mg/dL or HbA1c >6.5% was the secondary criteria (20). Meeting one of these criteria was sufficient for a diabetes diagnosis. In this study, the type of diabetes was not distinguished.

### Definition of sarcopenia

Sarcopenia was assessed by ALM/BMI, with disease thresholds set at <0.789 for men and <0.512 for women by the National Institutes of Health Foundation (21). ALM/BMI was calculated by dividing the sum of the lean tissue of the limbs by the body mass index (21). ALM can be obtained by dual-energy X-ray absorptiometry. Pregnant women and individuals over 300 pounds (136 kg) or taller than 6 feet 5 inches (198 cm) were excluded due to dual-energy X-ray absorptiometry limitations.

### Definition of insufficient physical activity

Insufficient physical activity was defined as engaging in less than 150 minutes per week of moderate-intensity exercise and less than 75 minutes per week of vigorous-intensity exercise. This criterion aligns with the 2008 Physical Activity Guidelines for Americans (22).

### Covariate

Data on age, gender, race, education, and family monthly poverty level were sourced from the NHANES demographic profile. Hypertension was identified by a systolic blood pressure ≥140 mmHg or diastolic pressure ≥90 mmHg, average of three measurements (23). Non-HDL cholesterol was calculated by subtracting high density lipoprotein from total cholesterol, and the diabetic control target was set at less than 130 mg/dL (24). The criterion for intake of dietary cholesterol was set at less than 300 mg per day (25). The recommended standard for dietary fiber intake is 14 grams per 1000 kilocalories (26). Blood VD levels were categorized into severe deficiency (<10 ng/mL), deficiency (10 to <20 ng/mL), insufficiency (20 to <30 ng/mL), and adequacy (≥30 ng/mL) (27). The data represented the sum of 25-hydroxyvitamin D2 and D3, with blood VD concentrations converted to the unit of 1 ng/mL equaling 2.5 nmol/L.Glasses of daily drinking was calculated by dividing the total glasses of annual drinking by 365.

### Statistical analysis

Fasting sample weights were incorporated to account for non-response, coverage gaps, and unequal selection probabilities across specific population categories, thereby facilitating the generation of national estimates for all analyses.

All independent variables and covariates included in the analysis were transformed into categorical variables. Statistical outcomes were reported as percentages with corresponding 95% confidence interval (CI). Categorical variables were examined using the chi-square test to assess statistical differences. To ensure the absence of multicollinearity, all covariates were evaluated, with a variance inflation factor (VIF) threshold set at less than 5. The population structures of diabetic and non-diabetic groups were analyzed, considering sarcopenia and insufficient physical activity as risk factors. Three models were developed to examine the relationship between the independent and dependent variables. Model 1 included solely the independent and dependent variables, without the inclusion of any covariates. Model 2 was adjusted for gender, age, and ethnicity. Building upon Model 2, Model 3 incorporated additional adjustments for education, family monthly poverty level, blood pressure, non-HDL, blood VD levels, dietary cholesterol, dietary fiber and glasses of daily drinking. Based on model 3, I performed two stratified analyses for non-diabetic and diabetic group in sarcopenia and insufficient physical activity respectively. The logistic regression outcomes from Model 3 were employed to evaluate the odds ratio (OR) and 95% confidence interval (CI) for variables related to the prevalence of diabetes. In this model, co-occurrence of sarcopenia and insufficient physical activity were analyzed for interaction with each covariate. The results of the logistic regression and interaction analysis from Model 3 were visualized using the “forest plot” feature in GraphPad Prism (version 8.0).

All statistical analyses were conducted using EmpowerStats (version 2.0) and STATA (version 17.0) software, while figures were generated with GraphPad Prism (version 8.0). P-value<0.05 was considered statistically significant.

## Result

### Participant characteristics

All results showed significant statistical differences except for gender, dietary fiber intake and glasses of daily drinking. Diabetes prevalence was strongly linked to age (P<0.001), with 68.4%(95%CI,62.6%-73.7%) of the sample being middle-aged (45-59 years) in the diabetic group, significantly higher than the 34.2% (95%CI,32.1%-36.3%) in the non-diabetic group. Ethnic differences significantly correlate with diabetes susceptibility: Mexican American (14.7%,95%CI,11.7%-18.3% vs 9.6%,95% CI,8.8%-10.5%)、Other Hispanic (9.7%,95%CI,7.4%-12.7% vs 6.9%,95%CI,6.2%-7.7%) and Non-Hispanic Black (16.0%,95% CI,13.0%-19.5% vs 11.4%,95%CI,10.5%-12.3%) are more prone, while Non-Hispanic White (50.0%,95%CI,43.9%-56.2% vs 62.8%,95%CI,61.0%-64.6%) are less prone. Diabetes risk was greater in individuals with lower education (47.3%,95% CI,41.2%-53.5% vs 31.3%,95%CI,29.5%-33.2%) compared to those with higher education (52.5%,95%CI,46.3%-58.6% vs 63.5%,95%CI,61.5%-65.5%). The percentage of diabetes group with a family poverty level of 1.30 (32.8%,95%CI,27.8%-38.3%) was higher than that of non-diabetic group (26.5%,95%CI,24.9%-28.2%).BMI had the strongest impact on diabetes prevalence (P<0.001). 66.1% (95%CI,60.2%-71.5%) of diabetics with a BMI ≥30kg/m^2^ compared to 32.7% (95%CI,30.%8-34.7%) of non-diabetes group. Hypertension was more common in diabetics (19.6%,95%CI,14.9%-25.4% vs 7.7%,95%CI,6.7%-8.9%), and non-HDL levels were more often elevated (65.5%,95%CI,59.6%-70.9% vs 51.5%,95%CI,49.4%-53.6%). VD deficiency leads to an increase in the prevalence of diabetes (35.6%,95%CI,29.9%-41.8% vs 22.6%,95%CI,21.1%-24.2%). More importantly, in diabetes group, the prevalence of sarcopenia (19.0%,95%CI,14.7%-24.1% vs 6.1%,95%CI,5.3%-7.0%) and insufficient physical activity (43.2%,95%CI,37.3%-49.4% vs 27.4%,95%CI,25.6%-29.3%) were significantly higher than those in non-diabetes group (P<0.001). The clinical demographic characteristics of all the 4255 participants included in the study are shown in **Table 1**.

**Table 1:** Characteristics of NHANES participants between 2011-2018 according to the presence of diabetes (n=4255).

Characteristics	Total (n=4255)	Diabetes		P-value
		No (n=3791)	Yes (n=464)	
**Gender**				0.080
Male	48.4 (46.4,50.4)	48.0 (45.9,50.1)	52.9 (46.7,59.0)	
Female	51.6 (49.6,53.6)	52.0 (49.9,54.1)	47.1 (41.0,53.3)	
**Age**				**<0.001**
18-44	63.0 (61.0,64.9)	65.8 (63.7,67.9)	31.6 (26.3,37.4)	
45-59	37.0 (35.1,39.0)	34.2 (32.1,36.3)	68.4 (62.6,73.7)	
**Race**				**<0.001**
Mexican American	10.0 (9.2,10.9)	9.6 (8.8,10.5)	14.7 (11.7,18.3)	
Other Hispanic	7.2 (6.5,7.9)	6.9 (6.2,7.7)	9.7 (7.4,12.7)	
Non-Hispanic White	61.7 (60.0,63.5)	62.8 (61.0,64.6)	50.0 (43.9,56.2)	
Non-Hispanic Black	11.8 (11.0,12.7)	11.4 (10.5,12.3)	16.0 (13.0,19.5)	
Non-Hispanic Asian	5.6 (5.1,6.1)	5.5 (5.0,6.1)	5.6 (4.1,7.5)	
Other Race	3.7 (3.1,4.5)	3.7 (3.0,4.6)	4.0 (2.4,6.5)	
**Education**				**<0.001**
≤High school	32.7 (30.9,34.5)	31.3 (29.5,33.2)	47.3 (41.2,53.5)	
>High school	62.6 (60.7,64.5)	63.5 (61.5,65.5)	52.5 (46.3,58.6)	
Not recorded	4.7 (4.1,5.5)	5.1 (4.4,6.0)	0.2 (0,0.8)	
**Family monthly poverty leve**l				**0.020**
≤1.30	27.1 (25.5,28.7)	26.5 (24.9,28.2)	32.8 (27.8,38.3)	
>1.3 to ≤1.85	12.0 (10.9,13.2)	12.2 (11.0,13.5)	9.6 (7.3,12.5)	
>1.85	56.9 (55.0,58.8)	57.1 (55.1,59.1)	55.3 (49.4,61.2)	
Not recorded	4.0 (3.4,4.8)	4.2 (3.5,5.0)	2.2 (1.1,4.5)	
**Body mass index (kg/m^2^)**				**<0.001**
<18.5	1.9 (1.4,2.5)	2.0 (1.5,2.6)	0.4 (0.1,1.8)	
≥18.5 to <25	30.8 (28.9,32.7)	32.8 (30.8,34.8)	8.9 (6.0,12.9)	
≥25 to <30	31.9 (30.0,33.8)	32.5 (30.6,34.5)	24.6 (19.8,30.1)	
≥30	35.5 (33.6,37.4)	32.7 (30.8,34.7)	66.1 (60.2,71.5)	
**Blood pressure (mmHg)**				**<0.001**
<140/90	88.5 (87.2,89.7)	89.7 (88.4,90.8)	76.0 (69.9,81.2)	
≥140/90	8.7 (7.7,9.9)	7.7 (6.7,8.9)	19.6 (14.9,25.4)	
Not recorded	2.8 (2.2,3.5)	2.6 (2.1,3.3)	4.4 (2.3,8.3)	
**non-HDL (mg/dL)**				**<0.001**
<130	46.7 (44.7,48.7)	47.8 (45.8,50.0)	33.8 (28.4,39.7)	
≥130	52.7 (50.7,54.6)	51.5 (49.4,53.6)	65.5 (59.6,70.9)	
Not recorded	0.7 (0.4,1.1)	0.7 (0.4,1.2)	0.7 (0.3,1.9)	
**Blood VD (ng/mL)**				**<0.001**
<10	3.0 (2.5,3.5)	3.0 (2.5,3.6)	2.8 (1.7,4.6)	
≥10 to <20	23.7 (22.2,25.3)	22.6 (21.1,24.2)	35.6 (29.9,41.8)	
≥20 to <30	40.1 (38.1,42.0)	40.6 (38.6,42.7)	33.8 (28.3,39.8)	
≥30	32.9 (31.0,34.9)	33.4 (31.4,35.6)	27.3 (22.1,33.2)	
Not recorded	0.3 (0.1,0.8)	0.3 (0.1,0.8)	0.4 (0.1,1.4)	
**Dietary fiber (g/1000kcal)**				0.224
<14	92.2 (91.1,93.2)	92.1 (90.9,93.1)	93.9 (90.9,95.9)	
≥14	7.8 (6.8,8.9)	7.9 (6.9,9.1)	6.1 (4.1,9.1)	
**Dietary cholesterol (mg/d)**				**<0.001**
<300	62.2 (60.2,64.1)	63.1 (61.1,65.1)	51.4 (45.2,57.6)	
≥300	37.8 (35.9,39.8)	36.9 (34.9,38.9)	48.6 (42.4,54.8)	
**Drink (glass/d)**				0.066
<1	72.8 (71.0,74.5)	72.4 (70.5,74.2)	76.7 (70.8,81.6)	
≥1 to <2	7.3 (6.3,8.4)	7.5 (6.5,8.8)	4.7 (2.7,7.9)	
≥2	6.3 (5.3,7.5)	6.5 (5.4,7.7)	4.3 (2.3,7.6)	
Not recorded	13.7 (12.5,15.0)	13.6 (12.4,14.9)	14.4 (10.3,19.7)	
**Sarcopenia**				**<0.001**
No	92.8 (91.9,93.7)	93.9 (93.0,94.7)	81.0 (75.9,85.3)	
Yes	7.2 (6.3,8.1)	6.1 (5.3,7.0)	19.0 (14.7,24.1)	
**Physical activity**				**<0.001**
Enough	71.3 (69.5,73.0)	72.6 (70.7,74.4)	56.8 (50.6,62.7)	
Not enough	28.7 (27.0,30.5)	27.4 (25.6,29.3)	43.2 (37.3,49.4)	

Categorical variables were expressed as a weighted percentage (95% confidence interval). Non-HDL, non-high density lipoprotein; VD, vitamin D. Statistically significant P-values are highlighted in bold type.

### Change in population structure

**Figure 2:**
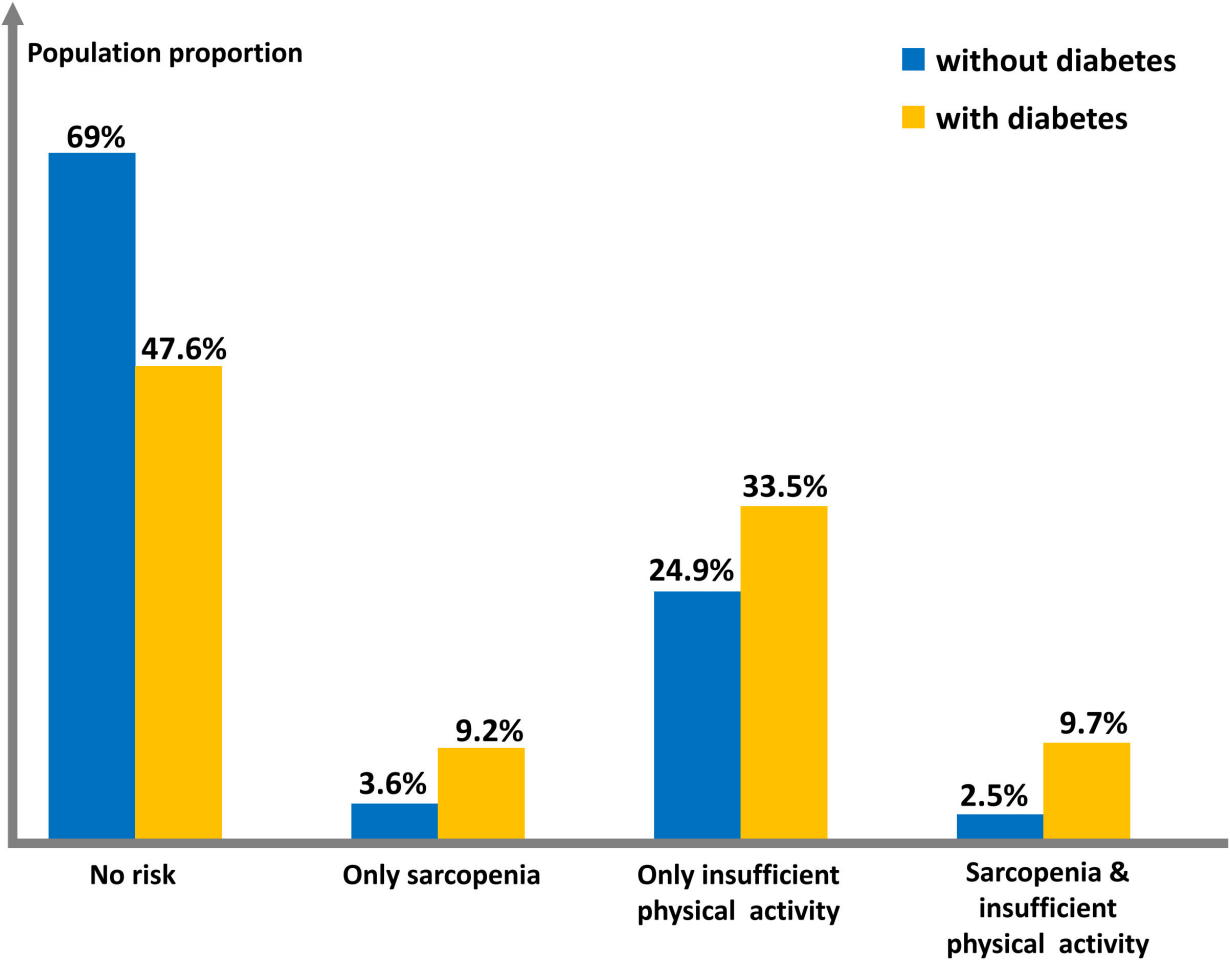
Histogram of population proportions characterized by sarcopenia and insufficient physical activity.

The study compared the prevalence of sarcopenia and insufficient physical activity between non-diabetic and diabetic populations. Data show a significant shift in the diabetic population’s structure (P<0.001), with a notably higher proportion having sarcopenia and/or insufficient physical activity, particularly when both are present, resulting in a 288% change rate. Refer to **Table 2** and **Figure 2** for details.

**Table 2:** Table of population proportions characterized by sarcopenia and insufficient physical activity.

Characteristics	Total (n=4255)	Without Diabetes (n=3791)	With Diabetes (n=464)	P-value
Risk factor				<0.001
No risk	67.2 (65.4,69.0)	69.0 (67.1,70.9)	47.6 (41.4,53.8)	
Only sarcopenia	4.1 (3.4,4.8)	3.6 (3.0,4.3)	9.2 (6.4,13.0)	
Only insufficient physical activity	25.6 (23.9,27.3)	24.9 (23.1,26.7)	33.5 (28.0,39.4)	
Sarcopenia & insufficient physical activity	3.1 (2.5,3.8)	2.5 (2.0,3.2)	9.7 (6.6,14.1)	

All values were expressed as a weighted percentage (95% confidence interval).

### Association between sarcopenia, insufficient physical activity and diabetes

Among the three models, both sarcopenia and insufficient physical activity were significant risk factors (P<0.05). In the fully adjusted model, having both risk factors increased diabetes risk by 1.45 times compared to having neither (OR=2.45,95% CI,1.35-4.44,P<0.05). This risk was higher than having only sarcopenia (OR=1.84,95%CI,1.09-3.11,P<0.05) or only insufficient physical activity (OR=1.55,95%CI,1.11-2.15,P<0.05) (**Table 3**).

**Table 3:** Correlation between sarcopenia, insufficient physical activity and diabetes.

Characteristics	Model 1		Model 2		Model 3	
	OR (95% CI)	P-value	OR (95% CI)	P-value	OR (95% CI)	P-value
No risk	Reference		Reference		Reference	
Only sarcopenia	3.72 (2.36,5.88)	<0.05	3.13 (1.87,5.24)	<0.05	1.84 (1.09,3.11)	<0.05
Only insufficient physical activity	1.95 (1.45,2.62)	<0.05	1.73 (1.26,2.38)	<0.05	1.55 (1.11,2.15)	<0.05
Sarcopenia &insufficient physical activity	5.66 (3.43,9.34)	<0.05	4.02 (2.34,6.92)	<0.05	2.45 (1.35,4.44)	<0.05

Model 1: No adjusted.

Model 2: Adjust for gender, age, and race.

Model 3: Adjusted for gender, age, race, education, family monthly poverty level, blood pressure, non-HDL, blood VD, dietary cholesterol, dietary fiber and glasses of daily drinking.

OR, odds ratio; 95% CI, 95% confidence interval.

The stratified analysis of Model 3, which focused on sarcopenia, indicated that younger age served as a protective factor within the nondiabetic cohort. This is attributed to the increased susceptibility to sarcopenia observed in the older age group (OR=1.70,95%CI,1.17-2.48,P=0.005). However, this protective effect was not evident in the diabetic cohort. Furthermore, the protective influences of race, education, and family monthly poverty level were also diminished in the presence of diabetes. The association between hypertension and sarcopenia was found to be significant in both groups, with a stronger correlation observed in the diabetic group (OR=2.58,95%CI,1.22-5.47,P=0.013) compared to the non-diabetic group (OR=1.64,95%CI,1.02-2.63,P=0.041). Regarding blood vitamin D levels, subgroups with insufficient (20 to <30ng/mL) and sufficient (≥30ng/mL) levels demonstrated a protective effect against sarcopenia in the non-diabetic group (OR=0.45,P=0.010 & OR=0.39,P=0.006, respectively). However, this protective effect was not observed in the diabetic group (**Table 4**).

**Table 4:** Stratified analysis of sarcopenia in non-diabetes group and diabetes group under model 3.

Characteristics	Without diabetes (n=3791)		With diabetes (n=464)	
	OR (95% CI)	P-value	OR (95% CI)	P-value
Gender				
Male	Reference		Reference	
Female	0.92 (0.65,1.29)	0.613	0.55 (0.28,1.09)	0.089
Age				
18-44	Reference		Reference	
45-59	**1.70 (1.17,2.48)**	**0.005**	0.92 (0.45,1.89)	0.828
Race				
Mexican American	Reference		Reference	
Other Hispanic	**0.63 (0.41,0.98)**	**0.040**	0.75 (0.31,1.84)	0.529
Non-Hispanic White	**0.35 (0.24,0.53)**	**<0.001**	**0.41 (0.19,0.88)**	**0.023**
Non-Hispanic Black	**0.08 (0.05,0.15)**	**<0.001**	**0.08 (0.03,0.24)**	**<0.001**
Non-Hispanic Asian	**0.48 (0.30,0.77)**	**0.002**	0.34 (0.11,1.02)	0.055
Other Race	**0.27 (0.10,0.71)**	**0.008**	**0.21 (0.05,0.94)**	**0.041**
Education				
≤High school	Reference		Reference	
>High school	**0.68 (0.47,0.99)**	**0.042**	1.08 (0.57,2.05)	0.803
Family monthly poverty level				
≤1.30	Reference		Reference	
>1.3 to ≤1.85	0.88 (0.54,1.42)	0.590	0.80 (0.31,2.09)	0.649
>1.85	**0.65 (0.43,0.98)**	**0.039**	0.90 (0.48,1.71)	0.750
Blood pressure (mmHg)				
<140/90	Reference		Reference	
≥140/90	**1.64 (1.02,2.63)**	**0.041**	**2.58 (1.22,5.47)**	**0.013**
non-HDL (mg/dL)				
<130	Reference		Reference	
≥130	**1.60 (1.12,2.30)**	**0.011**	1.03 (0.49,2.15)	0.944
Blood VD (ng/mL)				
<10	Reference		Reference	
≥10 to <20	0.72 (0.39,1.33)	0.294	1.56 (0.29,8.38)	0.603
≥20 to <30	**0.45 (0.24,0.83)**	**0.010**	1.49 (0.28,7.89)	0.642
≥30	**0.39 (0.20,0.77)**	**0.006**	1.73 (0.3,10.04)	0.542
Dietary fiber (g/1000kcal)				
<14	Reference		Reference	
≥14	0.81 (0.49,1.33)	0.395	0.35 (0.11,1.11)	0.074
Dietary cholesterol (mg/d)				
<300	Reference		Reference	
≥300	0.82 (0.57,1.16)	0.258	**0.43 (0.20,0.90)**	**0.026s**

Non-HDL, non-high density lipoprotein; VD, vitamin D; OR, odds ratio; 95% CI, 95% confidence interval. Statistically significant data are highlighted in bold type.

A stratified analysis of Model 3 based on insufficient physical activity revealed that female in the non-diabetic cohort exhibited a higher likelihood of insufficient physical activity (OR=2.10,95%CI,1.71-2.58,P<0.001), as did the elderly population aged 45-59 years (OR=1.69,95%CI,1.37-2.10,P<0.001). However, no such association was observed in the diabetic group. Within the non-diabetic group, elevated non-HDL cholesterol levels (≥130 mg/dL) were significantly correlated with insufficient physical activity (OR=1.44,95%CI,1.17-1.76,P<0.001). But no such association was found in the diabetes group. For blood vitamin D levels, similar to **Table 4**, the insufficient (20 to <30ng/mL) and sufficient (≥30ng/mL) subgroups showed a protective effect in the non-diabetic group (OR=0.55,P=0.011 & OR=0.37,P<0.001,respectively). Again, this effect was still absent in the diabetic group (**Table 5**).

**Table 5:** Stratified analysis of insufficient physical activity in non-diabetes group and diabetes group under model 3.

Characteristics	Without diabetes (n=3791)		With diabetes (n=464)	
	OR(95% CI)	P-value	OR(95% CI)	P-value
Gender				
Male	Reference		Reference	
Female	**2.10 (1.71,2.58)**	**<0.001**	1.69 (0.95,3.00)	0.074
Age				
18-44	Reference		Reference	
45-59	**1.69 (1.37,2.10)**	**<0.001**	1.28 (0.72,2.28)	0.408
Race				
Mexican American	Reference		Reference	
Other Hispanic	0.95 (0.70,1.31)	0.775	1.44 (0.69,3.02)	0.331
Non-Hispanic White	0.89 (0.68,1.17)	0.415	1.08 (0.55,2.11)	0.827
Non-Hispanic Black	0.76 (0.57,1.00)	0.052	0.98 (0.49,1.94)	0.950
Non-Hispanic Asian	1.25 (0.92,1.70)	0.147	1.74 (0.78,3.88)	0.174
Other Race	0.62 (0.37,1.04)	0.071	2.72 (0.90,8.18)	0.075
Education				
≤High school	Reference		Reference	
>High school	0.86 (0.69,1.07)	0.165	0.81 (0.45,1.45)	0.476
Family monthly poverty level				
≤1.30	Reference		Reference	
>1.3 to ≤1.85	1.11 (0.84,1.48)	0.462	1.14 (0.59,2.20)	0.696
>1.85	1.02 (0.82,1.27)	0.887	1.06 (0.59,1.92)	0.836
Blood pressure (mmHg)				
<140/90	Reference		Reference	
≥140/90	1.00 (0.71,1.41)	0.990	1.49 (0.73,3.05)	0.271
non-HDL (mg/dL)				
<130	Reference		Reference	
≥130	**1.44 (1.17,1.76)**	**<0.001**	0.91 (0.53,1.56)	0.726
Blood VD (ng/mL)				
<10	Reference		Reference	
≥10 to <20	0.74 (0.47,1.16)	0.189	0.50 (0.17,1.44)	0.198
≥20 to <30	**0.55 (0.35,0.87)**	**0.011**	0.71 (0.24,2.08)	0.530
≥30	**0.37 (0.23,0.61)**	**<0.001**	0.71 (0.23,2.23)	0.561
Dietary fiber (g/1000kcal)				
<14	Reference		Reference	
≥14	**0.59 (0.41,0.85)**	**0.004**	0.69 (0.30,1.59)	0.385
Dietary cholesterol (mg/d)				
<300	Reference		Reference	
≥300	0.85 (0.69,1.04)	0.111	0.90 (0.52,1.57)	0.718

Non-HDL, non-high density lipoprotein; VD, vitamin D; OR, odds ratio; 95% CI, 95% confidence interval. Statistically significant data are highlighted in bold type.

A more detailed stratified analysis of Model 3 revealed that the elevated risk of diabetes was more pronounced in individuals exhibiting both sarcopenia and insufficient physical activity. The increased risk of diabetes was observed in 13 subgroups among individuals exhibiting both sarcopenia and insufficient physical activity, compared to 11 subgroups among those with insufficient physical activity only, and 6 subgroups among those with sarcopenia only. When sarcopenia and insufficient physical activity co-occur, compared to no risk factors, the details are as follows. Among the uncontrollable factors, race had the most obvious effect on diabetes, Other Hispanic and Non-Hispanic White were more susceptible, with OR of 5.03 (95% CI,1.65-15.33,P=0.005) and 4.02 (95%CI,1.41-11.43,P=0.009), respectively. Next by gender, male’s OR was 3.90 (95% CI,1.56-9.71,P=0.004). Age also showed a clear difference, with an OR of people aged 45-59 years old of 2.28 (95% CI,1.08-4.85,P=0.032).Among the controllable factors, the blood pressure ≥140/90 mmHg was the most significant, with an OR of 6.67 (95%CI,1.78-24.92,P=0.005). This was followed by Blood VD ≥30 ng/mL with an OR of 5.10 (95%CI,1.65-15.71,P=0.005). Education also showed a clear effect, with the OR for those educated ≥High school being 3.88 (95%CI,1.66-9.06,P=0.002). Details are given in **Table 6**.

**Table 6:** Stratified analysis of the correlation between sarcopenia, insufficient physical activity and diabetes under model 3.

Characteristics	No risk (n=2744)	Only Sarcopenia (n=210)		Only insufficient physical activity (n=1142)		Sarcopenia & insufficient physical activity (n=159)	
		OR (95% CI)	P-value	OR (95% CI)	P-value	OR (95% CI)	P-value
Gender							
Male	Reference	**2.80 (1.48,5.30)**	**0.002**	**1.89 (1.15,3.12)**	**0.012**	**3.90 (1.56,9.71)**	**0.004**
Female	Reference	1.07 (0.42,2.74)	0.887	1.24 (0.80,1.92)	0.336	1.70 (0.71,4.07)	0.235
Age							
18-44	Reference	**3.14 (1.42,6.91)**	**0.005**	**1.89 (1.13,3.14)**	**0.015**	2.55 (0.96,6.78)	0.060
45-59	Reference	1.26 (0.67,2.37)	0.468	1.43 (0.94,2.18)	0.096	**2.28 (1.08,4.85)**	**0.032**
Race							
Mexican American	Reference	1.41 (0.62,3.25)	0.414	1.59 (0.80,3.17)	0.188	0.98 (0.38,2.57)	0.971
Other Hispanic	Reference	2.80 (0.72,10.89)	0.137	**2.59 (1.16,5.78)**	**0.020**	**5.03 (1.65,15.33)**	**0.005**
Non-Hispanic White	Reference	2.40 (0.96,5.99)	0.060	1.35 (0.73,2.49)	0.341	**4.02 (1.41,11.43)**	**0.009**
Non-Hispanic Black	Reference	2.00 (0.68,5.91)	0.211	1.27 (0.77,2.09)	0.353	0.69 (0.15,3.11)	0.624
Non-Hispanic Asian	Reference	1.34 (0.34,5.30)	0.672	2.18 (0.96,4.94)	0.064	1.74 (0.50,6.11)	0.387
Other Race	Reference	Not recorded		Not recorded		Not recorded	
Education							
≤High school	Reference	1.89 (1.00,3.59)	0.051	**1.92 (1.18,3.12)**	**0.008**	1.53 (0.77,3.02)	0.222
>High school	Reference	1.78 (0.73,4.35)	0.208	1.22 (0.77,1.92)	0.395	**3.88 (1.66,9.06)**	**0.002**
Family monthly poverty level							
≤1.30	Reference	1.52 (0.73,3.15)	0.263	**1.79 (1.12,2.85)**	**0.014**	2.36 (0.93,5.95)	0.069
>1.3 to ≤1.85	Reference	1.67 (0.52,5.29)	0.387	**2.64 (1.14,6.11)**	**0.023**	0.91 (0.13,6.51)	0.922
>1.85	Reference	2.28 (0.96,5.41)	0.062	1.28 (0.76,2.17)	0.352	**3.03 (1.20,7.67)**	**0.019**
Blood pressure (mmHg)							
<140/90	Reference	**1.93 (1.06,3.52)**	**0.032**	**1.59 (1.10,2.29)**	**0.013**	**2.01 (1.01,4.00)**	**0.048**
≥140/90	Reference	2.24 (0.65,7.72)	0.203	1.63 (0.77,3.42)	0.199	**6.67 (1.78,24.92)**	**0.005**
non-HDL (mg/dL)							
<130	Reference	**3.03 (1.29,7.09)**	**0.011**	**2.36 (1.35,4.13)**	**0.003**	3.53 (0.89,14.01)	0.073
≥130	Reference	1.51 (0.79,2.87)	0.214	1.24 (0.81,1.90)	0.325	**2.31 (1.15,4.66)**	**0.019**
Blood VD (ng/mL)							
<10	Reference	8.02 (0.55,118.01)	0.129	2.06 (0.33,12.69)	0.437	0.87 (0.10,7.86)	0.903
≥10 to <20	Reference	1.87 (0.88,3.96)	0.102	1.32 (0.76,2.30)	0.317	0.75 (0.29,1.92)	0.552
≥20 to <30	Reference	1.11 (0.45,2.71)	0.827	1.38 (0.78,2.44)	0.272	**3.68 (1.43,9.51)**	**0.007**
≥30	Reference	1.89 (0.54,6.66)	0.322	**1.97 (1.02,3.80)**	**0.043**	**5.10 (1.65,15.71)**	**0.005**
Dietary fiber (g/1000kcal)							
<14	Reference	**1.99 (1.16,3.41)**	**0.013**	**1.56 (1.10,2.21)**	**0.013**	**2.67 (1.45,4.95)**	**0.002**
≥14	Reference	0.56 (0.06,5.35)	0.616	1.46 (0.50,4.26)	0.490	0.68 (0.12,3.70)	0.654
Dietary cholesterol (mg/d)							
<300	Reference	**2.07 (1.02,4.20)**	**0.043**	1.44 (0.93,2.23)	0.099	**3.05 (1.43,6.51)**	**0.004**
≥300	Reference	1.72 (0.81,3.64)	0.155	**1.80 (1.08,3.01)**	**0.025**	1.83 (0.71,4.70)	0.207

Non-HDL, non-high density lipoprotein; VD, vitamin D; OR, odds ratio; 95% CI, 95% confidence interval. Statistically significant data are highlighted in bold type.

**Figure 3:**
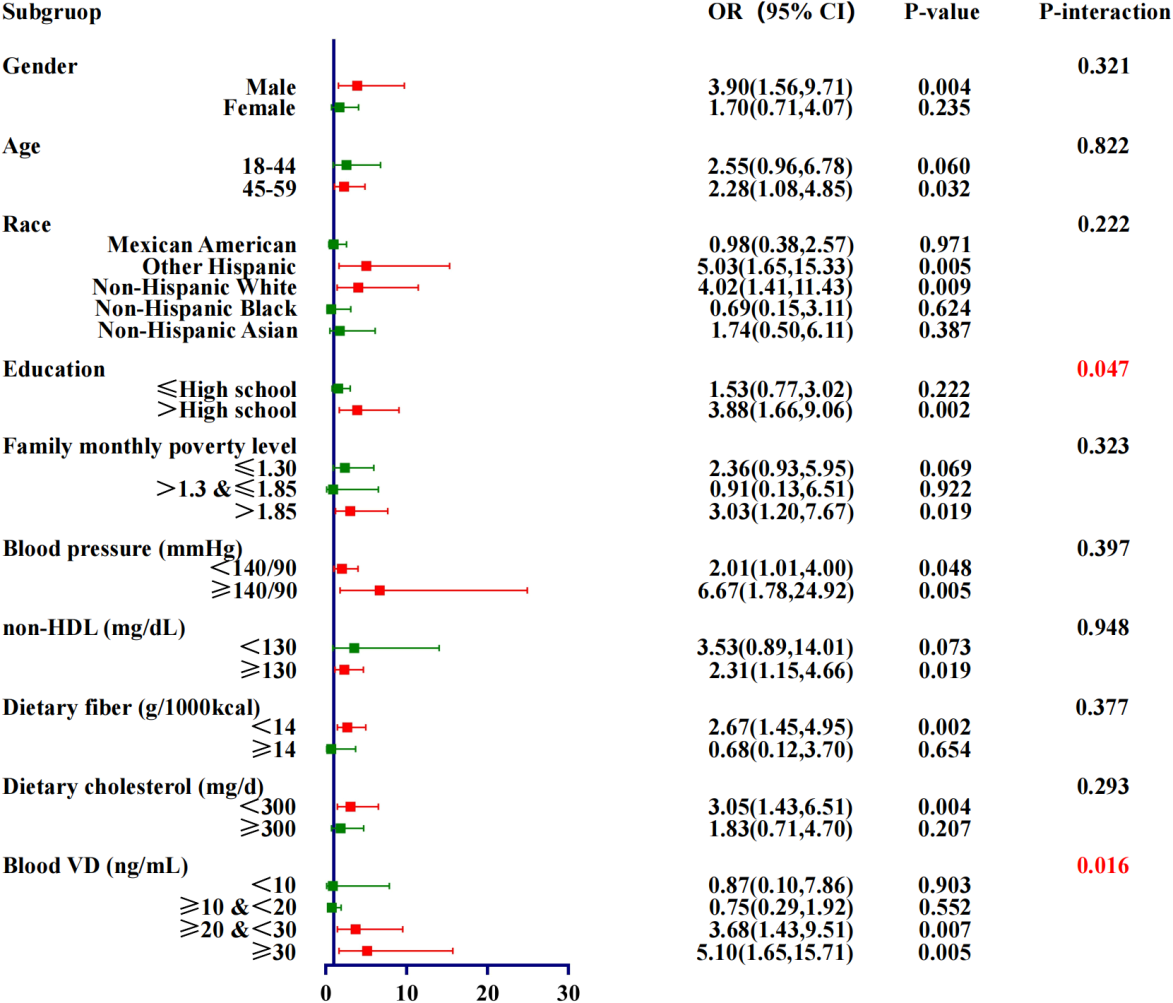
Forest plot for subgruop of sarcopenia & insufficient physical activity.

**Figure 3** displays the interaction analysis results of co-occurrence of sarcopenia and insufficient physical activity with each covariate, specifically blood VD (P=0.016) and education (P=0.047).

## Discussion

The global prevalence of diabetes is on the rise (1). Research indicates that sarcopenia and insufficient physical activity are significant risk factors for the development of diabetes (28). The reduction in skeletal muscle mass and strength associated with sarcopenia can adversely affect bodily function, a condition that becomes increasingly evident with advancing age (29). Furthermore, there exists a bidirectional relationship between diabetes and sarcopenia, as they share numerous common risk factors, including chronic inflammation and insulin resistance (29). Insufficient physical activity not only hastens the onset of sarcopenia (30) but also adversely impacts the management of diabetes. Empirical evidence indicates that appropriate exercise regimens, particularly those involving resistance training and aerobic activities, enhance muscle mass and strength in individuals with diabetes, thereby mitigating the risk associated with the condition (31). Furthermore, physical exercise contributes to blood glucose regulation by enhancing insulin sensitivity and reducing inflammatory markers (17). Consequently, interventions targeting individuals with diabetes ought to incorporate strategies aimed at enhancing muscle mass and encouraging physical activity, thereby mitigating the risk of sarcopenia and enhancing overall health outcomes (28, 29).

In this study, a significant synergistic effect was observed between sarcopenia and insufficient physical activity, resulting in a markedly higher risk of diabetes compared to the presence of either sarcopenia or insufficient physical activity alone (**Table 3**). This effect is particularly evident in the changes observed in the population structure. The population was categorized into four distinct groups: no risk, sarcopenia only, insufficient physical activity only, and both sarcopenia and insufficient physical activity. An analysis of the population structure of individuals with diabetes compared to those without reveals a significant disparity. Specifically, the prevalence of both sarcopenia and insufficient physical activity is notably higher among the diabetic population, with a prevalence rate that is 2.88 times greater than that of the non-diabetic population. Simultaneously, it is imperative to note that the prevalence of sarcopenia alone in diabetic group is 2.56 times greater than that observed in non-diabetic group, which is close to the prevalence of both sarcopenia and insufficient physical activity. In the stratified analysis (**Table 6**), six subgroups exhibited an increased risk when considering sarcopenia alone, which significantly overlapped with the presence of both sarcopenia and insufficient physical activity. Eleven subgroups exhibited an increased risk of onset when characterized solely by insufficient physical activity; however, only five subgroups overlapped with the concurrent presence of both sarcopenia and insufficient physical activity. Analyses of population structure and stratified results indicated that both sarcopenia and insufficient physical activity contribute to the onset of diabetes, albeit through distinct mechanisms (12, 16). Figuratively, if one were to visualize these factors as a three-dimensional construct, the impact of sarcopenia could be likened to depth, while insufficient physical activity would represent breadth. In conclusion, the simultaneous presence of both conditions further exacerbates the risk of developing diabetes.

Previous research has established individual associations between sarcopenia, insufficient physical activity and diabetes. However, the combined impact of the two factors on the risk of developing diabetes has not been thoroughly examined. Building upon prior studies, this investigation explores the concurrent presence of sarcopenia and insufficient physical activity. The findings align with the majority of existing literature, indicating a significant increase in diabetes risk when both risk factors are present. Nonetheless, two key aspects require further clarification. One aspect under investigation is the impact of blood VD levels on the onset of diabetes. While previous research has identified VD deficiency as a potential risk factor for diabetes (32), this study presents contrasting findings. Specifically, the study found no increased risk of diabetes onset associated with VD deficiency (10 to <20 ng/mL) or severe deficiency (<10 ng/mL). Conversely, the risk was significantly elevated when VD levels were insufficient (20 to <30 ng/mL) or even sufficient (≥30 ng/mL), with OR of 3.68 (P=0.007) and 5.10 (P=0.005), respectively. My understanding is that in the study of diabetes onset risk, previous studies did not include sarcopenia and insufficient physical activity in the analysis, but only analyzed the association between blood VD and diabetes (33). This suggests that existing studies may overlook other important factors affecting diabetes risk, such as muscle mass and physical activity level. The relationship between sarcopenia and diabetes has not been fully explored, and insufficient physical activity is considered as an important factor leading to sarcopenia and metabolic disorders (34). Blood VD serves as a protective factor against diabetes; however, its protective efficacy is subject to a threshold (35). When sarcopenia and insufficient physical activity co-occur and reach a critical intensity, the protective effect of VD diminishes, resulting in an elevated risk of diabetes. This interaction effect among the three factors has been statistically validated (P-interaction=0.016).Another factor to consider is the influence of education on the onset of diabetes. Specifically, in relation to diabetes onset risk, higher levels of education, in conjunction with sarcopenia and insufficient physical activity, exhibit a synergistic effect that elevates the risk of developing diabetes (P-interaction=0.047). Individuals with higher educational attainment are statistically more likely to develop diabetes (OR=3.88,P=0.002). This increased risk may be attributed to their propensity to engage in sedentary occupations, which are associated with prolonged periods of inactivity. Such lifestyle patterns can lead to the development of sarcopenia and insufficient physical activity, thereby contributing to an elevated risk of diabetes (36).

### Strengths of this study

This study investigates the combined impact of sarcopenia and insufficient physical activity on the onset of diabetes, thereby supplementing existing research. Through an analysis of population structure, the study elucidates the influence of sarcopenia and insufficient physical activity by examining changes in population proportions.

### Limitations of this study

The NHANES database does not include data on body composition for pregnant women and individuals aged ≥60 years, rendering it inadequate for studying these populations. Additionally, as a cross-sectional survey, the NHANES database is limited in its ability to establish causal inferences. Consequently, it is imperative to develop specific methodologies for assessing body composition in pregnant women and individuals aged 60 years, coupled with long-term follow-up studies, to facilitate more comprehensive research. Self-report bias is unavoidable in physical activity assessments using questionnaires. Additionally, the participant data lacked precise timing for lab tests, preventing the elimination of seasonal effects on blood vitamin D levels.

## Conclusion

The simultaneous occurrence of sarcopenia and insufficient physical activity markedly elevates the risk of developing diabetes, demonstrating a synergistic interaction between these two factors. Therefore, it is imperative to conduct concurrent screening for sarcopenia and evaluation of physical activity levels as a preventative measure against diabetes.

## Data Availability

The original contributions presented in the study are included in the article/supplementary material. Further inquiries can be directed to the corresponding author.

## References

[1] Sun H Saeedi P Karuranga S Pinkepank M Ogurtsova K Duncan BB. IDF Diabetes Atlas: Global, regional and country-level diabetes prevalence estimates for 2021 and projections for 2045. Diabetes Res Clin Pract. (2022) 183:109119. doi: 10.1016/j.diabres.2021.109119 34879977 PMC11057359

[2] Bloomgarden ZT. Diabetes and cardiovascular disease. Diabetes Care. (2011) 34:e24–30. doi: 10.2337/dc11-0007 PMC3041229 21357355

[3] Kikuchi K Saigusa D Kanemitsu Y Matsumoto Y Thanai P Suzuki N. Gut microbiome-derived phenyl sulfate contributes to albuminuria in diabetic kidney disease. Nat Commun. (2019) 10:1835. doi: 10.1038/s41467-019-09735-4 31015435 PMC6478834

[4] Yan T Venkat P Chopp M Zacharek A Ning R Cui Y. Neurorestorative therapy of stroke in type 2 diabetes mellitus rats treated with human umbilical cord blood cells. Stroke. (2015) 46:2599–606. doi: 10.1161/STROKEAHA.115.009870 PMC4550506 26243222

[5] Bhatia HS Brunner AD Ozturk F Kapoor S Rong Z Mai H. Spatial proteomics in three-dimensional intact specimens. Cell. (2022) 185:5040–58 e19. doi: 10.1016/j.cell.2022.11.021 36563667

[6] Centor RM Laiteerapong N Winn AN. Web exclusive. Annals on call - first-line drug therapy for type 2 diabetes. Ann Intern Med. (2023) 176:eA220004. doi: 10.7326/A22-0004 36802895

[7] Alhaddad AY Aly H Gad H Al-Ali A Sadasivuni KK Cabibihan JJ. Sense and learn: recent advances in wearable sensing and machine learning for blood glucose monitoring and trend-detection. Front Bioeng Biotechnol. (2022) 10:876672. doi: 10.3389/fbioe.2022.876672 35646863 PMC9135106

[8] Muntean C Starcea IM Banescu C. Diabetic kidney disease in pediatric patients: A current review. World J Diabetes. (2022) 13:587–99. doi: 10.4239/wjd.v13.i8.587 PMC9412860 36159227

[9] Rosenberg IH. Sarcopenia: origins and clinical relevance. Clin Geriatr Med. (2011) 27:337–9. doi: 10.1016/j.cger.2011.03.003 21824550

[10] Cruz-Jentoft AJ Bahat G Bauer J Boirie Y Bruyere O Cederholm T. Sarcopenia: revised European consensus on definition and diagnosis. Age Ageing. (2019) 48:601. doi: 10.1093/ageing/afz046 PMC6593317 31081853

[11] Livshits G Kalinkovich A. Inflammaging as a common ground for the development and maintenance of sarcopenia, obesity, cardiomyopathy and dysbiosis. Ageing Res Rev. (2019) 56:100980. doi: 10.1016/j.arr.2019.100980 31726228

[12] Nedergaard A Sun S Karsdal MA Henriksen K Kjaer M Lou Y. Type VI collagen turnover-related peptides-novel serological biomarkers of muscle mass and anabolic response to loading in young men. J Cachexia Sarcopenia Muscle. (2013) 4:267–75. doi: 10.1007/s13539-013-0114-x PMC3830008 23943593

[13] Kim TN Park MS Yang SJ Yoo HJ Kang HJ Song W. Prevalence and determinant factors of sarcopenia in patients with type 2 diabetes: the Korean Sarcopenic Obesity Study (KSOS). Diabetes Care. (2010) 33:1497–9. doi: 10.2337/dc09-2310 PMC2890348 20413515

[14] Li Y Wang DD Ley SH Vasanti M Howard AG He Y. Time trends of dietary and lifestyle factors and their potential impact on diabetes burden in China. Diabetes Care. (2017) 40:1685–94. doi: 10.2337/dc17-0571 PMC5862128 29046327

[15] Mohanty SA Woolhandler S Himmelstein DU Bor DH. Diabetes and cardiovascular disease among Asian Indians in the United States. J Gen Intern Med. (2005) 20:474–8. doi: 10.1111/j.1525-1497.2005.40294.x PMC1490101 15963176

[16] Bergman RN Piccinini F Kabir M Kolka CM Ader M. Hypothesis: role of reduced hepatic insulin clearance in the pathogenesis of type 2 diabetes. Diabetes. (2019) 68:1709–16. doi: 10.2337/db19-0098 PMC6702636 31431441

[17] Lu Z Hu Y He H Chen X Ou Q Liu Y. Associations of muscle mass, strength, and quality with diabetes and the mediating role of inflammation in two National surveys from China and the United states. Diabetes Res Clin Pract. (2024) 214:111783. doi: 10.1016/j.diabres.2024.111783 39002932

[18] Divney AA Murillo R Rodriguez F Mirzayi CA Tsui EK Echeverria SE. Diabetes prevalence by leisure-, transportation-, and occupation-based physical activity among racially/ethnically diverse U. S. Adults. Diabetes Care. (2019) 42:1241–7. doi: 10.2337/dc18-2432 PMC6609958 31221695

[19] NHANES-national health and nutrition examination survey homepage. Available online at: https://www.cdc.gov/nchs/nhanes (Accessed December 27, 2024).

[20] ElSayed NA Aleppo G Aroda VR Bannuru RR Brown FM Bruemmer D. 2. Classification and diagnosis of diabetes: standards of care in diabetes-2023. Diabetes Care. (2023) 46:S19–40. doi: 10.2337/dc23-S002 PMC9810477 36507649

[21] Studenski SA Peters KW Alley DE Cawthon PM McLean RR Harris TB. The FNIH sarcopenia project: rationale, study description, conference recommendations, and final estimates. J Gerontol A Biol Sci Med Sci. (2014) 69:547–58. doi: 10.1093/gerona/glu010 PMC3991146 24737557

[22] Araujo J Cai J Stevens J. Prevalence of optimal metabolic health in American adults: national health and nutrition examination survey 2009-2016. Metab Syndr Relat Disord. (2019) 17:46–52. doi: 10.1089/met.2018.0105 30484738

[23] Whelton PK Carey RM Aronow WS Casey DE Jr. Collins KJ Dennison Himmelfarb C. 2017 ACC/AHA/AAPA/ABC/ACPM/AGS/APhA/ASH/ASPC/NMA/PCNA guideline for the prevention, detection, evaluation, and management of high blood pressure in adults: A report of the American college of cardiology/American heart association task force on clinical practice guidelines. J Am Coll Cardiol. (2018) 71:e127–248. doi: 10.1016/j.jacc.2017.11.006 29146535

[24] National Center for Chronic Disease Prevention and Health Promotion (U.S.). Division of Diabetes Translation. National diabetes statistics report, 2020. Atlanta: Centers for Disease Control and Prevention (2020). Available at: https://stacks.cdc.gov/view/cdc/85309 (Accessed December 27, 2024).

[25] USDA and U.S. Department of Health and Human Services. Dietary Guidelines for Americans, 2010. 7th ed. Washington, DC: U.S. Government Printing Office (2010). Available at: https://odphp.health.gov/sites/default/files/2020-01/DietaryGuidelines2010.pdf (Accessed December 27, 2024). 10.3945/an.111.000430 PMC3090168 22332062

[26] Ionita-Mindrican CB Ziani K Mititelu M Oprea E Neacsu SM Morosan E. Therapeutic benefits and dietary restrictions of fiber intake: A state of the art review. Nutrients. (2022) 14:2641. doi: 10.3390/nu14132641 35807822 PMC9268622

[27] Holick MF. Vitamin D deficiency. N Engl J Med. (2007) 357:266–81. doi: 10.1056/NEJMra070553 17634462

[28] Hashimoto Y Takahashi F Okamura T Hamaguchi M Fukui M. Diet, exercise, and pharmacotherapy for sarcopenia in people with diabetes. Metabolism. (2023) 144:155585. doi: 10.1016/j.metabol.2023.155585 37156410

[29] Mesinovic J Fyfe JJ Talevski J Wheeler MJ Leung GKW George ES. Type 2 diabetes mellitus and sarcopenia as comorbid chronic diseases in older adults: established and emerging treatments and therapies. Diabetes Metab J. (2023) 47:719–42. doi: 10.4093/dmj.2023.0112 PMC10695715 37709502

[30] Golabi P Gerber L Paik JM Deshpande R de Avila L Younossi ZM. Contribution of sarcopenia and physical inactivity to mortality in people with non-alcoholic fatty liver disease. JHEP Rep. (2020) 2:100171. doi: 10.1016/j.jhepr.2020.100171 32964202 PMC7490851

[31] Peiris C. Supervised aerobic and resistance exercise improves glycaemic control and modifiable cardiovascular risk factors in people with Type 2 diabetes mellitus. J Physiother. (2011) 57:126. doi: 10.1016/S1836-9553(11)70024-8 21684495

[32] Tsur A Feldman BS Feldhammer I Hoshen MB Leibowitz G Balicer RD. Decreased serum concentrations of 25-hydroxycholecalciferol are associated with increased risk of progression to impaired fasting glucose and diabetes. Diabetes Care. (2013) 36:1361–7. doi: 10.2337/dc12-1050 PMC3631845 23393216

[33] Vinceti M Filippini T Wise LA Rothman KJ. A systematic review and dose-response meta-analysis of exposure to environmental selenium and the risk of type 2 diabetes in nonexperimental studies. Environ Res. (2021) 197:111210. doi: 10.1016/j.envres.2021.111210 33895112

[34] Tecilazich F Formenti AM Giustina A. Role of vitamin D in diabetic retinopathy: Pathophysiological and clinical aspects. Rev Endocr Metab Disord. (2021) 22:715–27. doi: 10.1007/s11154-020-09575-4 PMC7538371 33026598

[35] Corcoy R Mendoza LC Simmons D Desoye G Adelantado JM Chico A. The DALI vitamin D randomized controlled trial for gestational diabetes mellitus prevention: No major benefit shown besides vitamin D sufficiency. Clin Nutr. (2020) 39:976–84. doi: 10.1016/j.clnu.2019.04.006 31053513

[36] Diaz KM Howard VJ Hutto B Colabianchi N Vena JE Safford MM. Patterns of sedentary behavior and mortality in U.S. Middle-aged and older adults: A national cohort study. Ann Intern Med. (2017) 167:465–75. doi: 10.7326/M17-0212 PMC5961729 28892811

